# GPS Measurement Error Gives Rise to Spurious 180° Turning Angles and Strong Directional Biases in Animal Movement Data

**DOI:** 10.1371/journal.pone.0005632

**Published:** 2009-05-20

**Authors:** Amy Hurford

**Affiliations:** Centre for Mathematical Biology, Department of Biological Sciences, University of Alberta, Kingston, Ontario, Canada; University of Bristol, United Kingdom

## Abstract

**Background:**

Movement data are frequently collected using Global Positioning System (GPS) receivers, but recorded GPS locations are subject to errors. While past studies have suggested methods to improve location accuracy, mechanistic movement models utilize distributions of turning angles and directional biases and these data present a new challenge in recognizing and reducing the effect of measurement error.

**Methods:**

I collected locations from a stationary GPS collar, analyzed a probabilistic model and used Monte Carlo simulations to understand how measurement error affects measured turning angles and directional biases.

**Results:**

Results from each of the three methods were in complete agreement: measurement error gives rise to a systematic bias where a stationary animal is most likely to be measured as turning 180° or moving towards a fixed point in space. These spurious effects occur in GPS data when the measured distance between locations is <20 meters.

**Conclusions:**

Measurement error must be considered as a possible cause of 180° turning angles in GPS data. Consequences of failing to account for measurement error are predicting overly tortuous movement, numerous returns to previously visited locations, inaccurately predicting species range, core areas, and the frequency of crossing linear features. By understanding the effect of GPS measurement error, ecologists are able to disregard false signals to more accurately design conservation plans for endangered wildlife.

## Introduction

The number of animal movement studies that use data collected by Global Positioning System (GPS) receivers and other forms of radio telemetry has steadily increased in recent years. GPS data is frequently used to parameterize movement models to understand how small scale movement decisions give rise to larger scale patterns; particularly as individual-based movement models [1] or diffusion models [2]. Such mechanistic models can be used to determine how changes in the biotic or abiotic environment will affect the home ranges or geographic ranges of animals [3]. GPS technology has been applied to a wide range of taxa including mammals [4]–[6], reptiles [7], [8], fish [9], and birds [10]–[12]. Animal locations recovered from GPS receivers are subject to measurement error that can bias the parameterization of movement models [13]–[15] and give rise to patterns that could falsely be interpreted as biological signals [14], [16]. The implications of these errors can be profound because many conservation plans for endangered wildlife are based on habitat use patterns derived from movement data [17].

**Figure 1:**
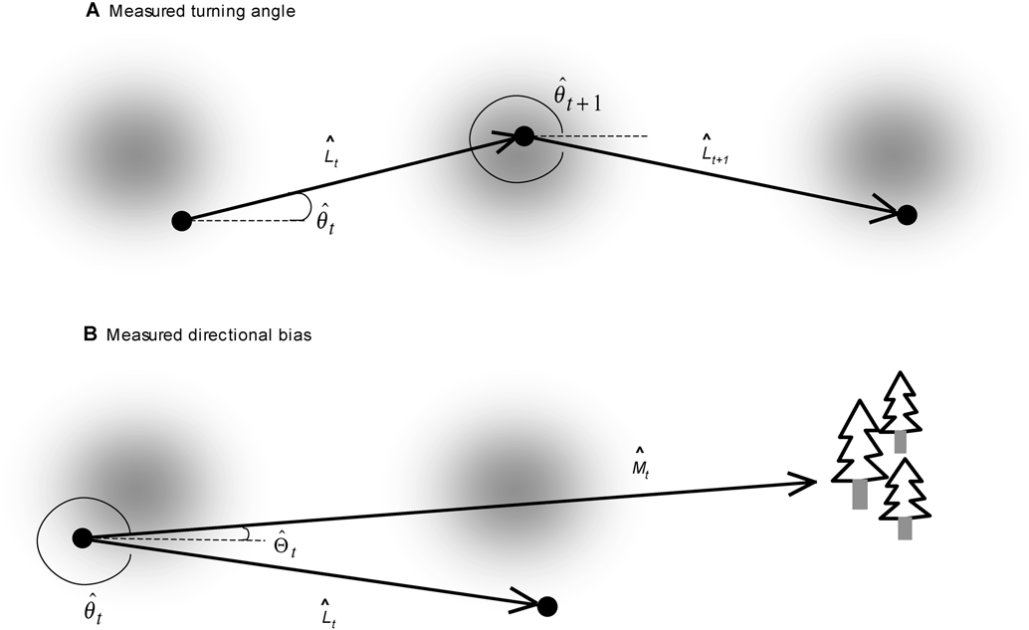
Location error can greatly affect (A) measured turning angles and (B) directional biases. The grey circles denote the probability density of measured locations for a moving animal. These are centered around the animal's true location. The solid black dots are measured locations. (A) The animal's true turning angle is zero, but the measured turning angle, 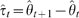, is approximately 45°. (B) The animal is moving towards the trees (true directional bias is 0°), however, because of measurement error in the animal's two measured locations the measured directional bias, 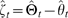, is approximately 315°.

Mechanistic movement models make assumptions concerning an animal's movement direction with reference to either internal or external factors. The correlated random walk model [18] allows for future movement direction to depend on past movement directions (i.e. an internal factor). The Fokker-Planck equation makes assumptions about an animal's movement with reference to an external bias point, i.e. the animal's den. These models suggest two important quantities that can be calculated from movement data: turning angles and directional biases. A turning angle is the difference in direction for two successive moves [19] (Figure 1A). A directional bias is the difference between the direction of animal movement and the direction of a hypothesized bias point [3] (Figure 1B). A measured GPS receiver location may differ from the true receiver location because of measurement error. This error in measured location then affects measured turning angles and directional biases (Figure 1). GPS data recovered from animals will contain multiple observations of turning angles and directional biases and can be summarized as a frequency histogram.

Most often animals will move in a straight line and movement data will show a high frequency of 0° turning angles [19]. Observations of 180° turning angles, indicating direction reversals, are less reported although searching behaviors may be associated with 180° turning angles. For example, in northern spotted owls (*Strix occidentalis caurina*) 180° turning angles may represent long distance exploratory forays ([19], Sec. 7.4), insects may show 180° turning angles when searching for food [20] and butterflies (*Euphydryas anicia*) may move with 180° turning angles when moving within a habitat patch [21]. A high frequency of direction reversals was reported for radio telemetry data from sedentary woodland caribou (*Rangifer tarandus*) [22]. While animal behavior may explain 180° turning angles, turning angles may be off by up to 160° when an animal moves a small distance and the accuracy of measured locations is poor [14]. In this paper, I will show that spurious direction reversal can arise due to measurement error in location.

There are a number of reasons why GPS locations are inaccurate [23]. Position Dilution of Precision (PDOP) value and fix status are proxies for the quality of GPS locations. Inaccurate locations are more likely when PDOP values are large indicating poor satellite geometry for triangulation between the GPS receiver and three or more GPS satelittes. If only three satellite signals are received, the GPS receiver will forego calculating its height [24] leading to a less accurate location [25]. This is referred to as a 2-D fix. Locations where all three spatial coordinates are estimated are referred to as 3-D fixes. Accuracy of GPS locations is improved using differential correction [24] which involves calibration of measured locations using the measured location of a base station relative to its true location. The accuracy of measured GPS locations is often improved by removing locations with high PDOP and/or 2-D fix status [25]–[29].

Previous studies provide some information on the effectiveness of GPS location filtering methods, however, assessment of their costs and benefits often assumes the research objective is to accurately describe the animal's movement path [25]–[27], [30] and not to accurately assess the distribution of measured turning angles. While filtering methods to improve the accuracy of measured locations may also improve the accuracy of measured turning angles and/or directional biases, the best methods for each will not be the same. Furthermore, different quantities, i.e. step length (the distance between successive locations), are relevant to assessing the quality of measured turning angles [15] but have little relevance to location accuracy.

Currently, there is no discussion of 180° turning angles arising in GPS data as a result of measurement error and consequently, it is not known whether recorded 180° turning angles can be attributed to animal behavior (i.e. searching) or not. This may lead to poor decision making when assessing the spatial accuracy necessary to address research questions relating to movement angles and to false interpretation of 180° turning angles as a type of searching or returning behavior.

In this manuscript, I use GPS data recovered from a gray wolf (*Canis lupus*) as a motivating case study that illustrates the prevalence of 180° turning angles in movement data. I demonstrate this pattern is consistent with the pattern caused by measurement error. I perform an experiment with a GPS collar that was stationary. In this experiment, the true turning angle and directional bias is undefined, however, in the presence of GPS measurement error successive measured locations were not identical and turning angles and directional biases could be calculated. I derive a probabilistic model for a stationary GPS receiver where I assume measurement error in location is a random variable drawn from a bivariate Normal distribution. I use several changes of variables to determine the probability density of measured turning angles and directional biases. Finally, I perform a computer simulation where receiver locations were simulated in the presence of GPS measurement error and I determine the relative frequency histogram of turning angles and directional biases over a range of different step lengths.

## Methods

Throughout this manuscript I denote measured values by the presence of a hat (∧) and the absence of a hat indicates a true location, direction, or angle. Measured turning angles were calculated as the difference in measured direction for two successive moves,
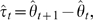
(1)where 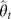 is the measured direction of movement at time *t*. The measured directional bias is the difference between the measured movement direction and the measured direction of the hypothesized bias point,
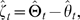
(2)where 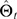 is the measured direction of the bias point at time *t*.

### 1) Case study: wolf movement data

I collected GPS data every 15 minutes from a gray wolf in northeastern Banff National Park and adjacent lands near Ya Ha Tinda Ranch, Alberta, Canada, from January 2–February 20, 2004. The data was collected using a Lotek 3300 GPS collar (Lotek Wireless Inc., Newmarket, Ontario, Canada). Elevation in the study area ranges between approximately 1500 and 3500 meters above sea level. Using these data, I calculated turning angles and directional biases using Equations 1 and 2 where the hypothesized bias point was assumed to be the centroid of the recorded locations. Measured step length (the measured distance between successive locations) was also calculated.

### 2) Stationary GPS collar experiment

I collected GPS locations every two hours from a stationary Lotek 2200 GPS collar that was hung from a rope tied between two iron stakes at a height of approximately 1 meter. The collar was located near the top of a southwest facing slope in mixed open conifer forest near Ya Ha Tinda, Alberta, Canada (605743 UTM Easting, 5727638 UTM Northing, North American Datum 1983, Zone 11) between March 30 and April 30, 2003. From the data recovered, I calculated measured turning angles and directional biases where the bias point was assumed to be the centroid of the recorded 3-D fix locations. I used the V test [31] with 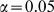 to test if the mean of the measured turning angles was 180° and if the mean of the measured directional biases was 0°.

I used data from this experiment to estimate the distribution of GPS measurement error. For each measured location, I calculated the displacement between the true collar location (assumed to be the centroid of the recorded 3-D fix locations) and the measured location. The distributions of measurement error that I fit to the stationary GPS collar data were the Normal distribution, 
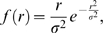
(3)the Laplace distribution,
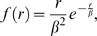
(4)and the Bessel distribution,
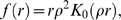
(5)where 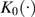 is the modified Bessel function of the second kind. For each distribution *f(r)* is the probability of a measured location observed at a Euclidean distance *r* from the true location. I estimated the parameters *σ*, *β* and *ρ* using maximum likelihood and determined the probability density function that best approximated the data by comparing the Akaike Information Criteria (AIC) values of competing models [32], [33].

### 3) Probabilistic model for a stationary GPS collar

I modeled measured GPS locations as an independent identically distributed random variable drawn from a radially symmetric bivariate Normal distribution centered at the receiver's true location. I selected the bivariate Normal distribution because it was analytically tractable for this problem. I used several change of variables to determine the distribution of measured turning angles. An identical method is used in [34] to understand the relationship between home range shapes on the angle and distance of successive moves. Full details of this procedure are found in Text S1.

### 4) Stochastic computer simulation model for a moving GPS collar

I used a stochastic simulation to determine the effect of step length on the distribution of measured turning angles and directional biases. I used error standard deviations as a measure of displacement (described in [15]), by dividing the step length by the standard deviation of the measurement error distribution to produce a non-dimensional measure of displacement.

I defined true animal locations using the true turning angle and the true step lengths. I assumed the animal's true turning angle was 0° and the true step length was the same on the first and second move. I repeated the simulation for every true step length from 0 to 1.35 error standard deviations by 6.7×10^−4^ error standard deviations.

I performed a Monte Carlo simulation where error was added to the true animal locations. I assumed the distribution of measurement error was the most parsimonious distribution from 2). I simulated measurement error using the inverse cumulative method to draw measured displacements from the measurement error distribution. This displacement was the displacement from the receiver's true location to the receiver's measured location. I determined the direction from the true location to the measured location by randomly drawing an angle from a uniform distribution on [0°, 360°). I simulated one hundred measured turning angles for each true step length and performed the V test [31] with 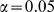 to determine if the turning angles were distributed unimodally with mean equal to the true turning angle of 0°. I repeated this procedure for a true turning angle of 90°.

For the directional bias computer simulations, I assumed a true directional bias of 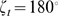 and generated one hundred measured directional biases for each step length from 0 to 1.35 error standard deviations by 6.7×10^−4^ and for *M* = 0.34 error standard deviations where *M* is the true distance from the collar's location to the bias point. I repeated this procedure for a true directional bias of 90°.

For stationary animals, I compared the results of 2), 3) and 4). I determined the proportion of measured turning angles on each 15° interval from 0–360° for 2) and 4). I compared this to the proportion of turning angles for the same 15° interval (by mid-point approximation) for 3). These comparisons test the sensitivity of the results to the assumptions made on the distribution of GPS measurement error.

### 5) Filtering of GPS data

I removed GPS locations from the stationary collar data using three of the filters suggested by [26]: removing locations with 1) PDOP>5, 2) PDOP>2 and 3) 2-D fix status. I assessed the accuracy of filtering methods by calculating the mean displacement from the measured locations to the true location (defined as the centroid of the 3-D fixes) and calculating the 50, 95, and 100 circular error probable (CEP; the radius of a circle that incorporates the specified percentile of locations [27], [28]). As in [26], I log-transformed these displacements, calculated the mean (or CEP) and then inverse transformed the results (see [31]). The cost of each filtering method is reported as the percentage of data removed or as the fraction of the original number of turning angles and directional biases that can be calculated from the remaining data. I also calculated the direction of the error in measured location for each of the filtering methods and used the Raleigh test [31] to test for a bias in the measured direction of the error. Of the three filtering methods, I applied the filtering method that provided the biggest improvement in accuracy with the least data reduction to the GPS data recovered from the gray wolf.

I used the results of 4) to suggest a threshold value that could be used to filter the wolf data using measured step lengths. I determined the minimum true step length where the true turning angle (0°) or directional bias (180°) was detected by the V test [31]. This is referred to as the true step length cutoff. I simulated 10000 measured step lengths where the true step length was assumed to be the true step length cutoff and determined the value of the 50^th^, 75^th^ and 95^th^ percentiles of measured step lengths. I assumed the displacement from the true receiver location to the measured receiver location was a Bessel distribution with ρ = 0.2 to 0.5 by 0.05. The true turning angles and directional biases for this simulation were set to 0° and 180° respectively as a worst case scenario since these signals require the largest true step length to be detected in the presence of error.

## Results

### Distribution of measurement error from the stationary GPS collar

For the stationary GPS collar experiment, 421 locations were recorded with a 96% fix rate. Removing locations with PDOP>2 (n = 227) or 2-D fix status (n = 233) yielded the most accurate estimates of collar location but eliminated over 50% of the measured locations and decreased the number of turning angles that could be calculated by near 75% (Tables 1 and 2). Removing locations with PDOP>5 (n = 32) was judged the best filtering method as it improved accuracy with minimal loss of data (Tables 1 and 2). Results of the Raleigh test showed that the errors in measured locations were not consistently in any particular direction (Table 2). The centroid of 3-D fixes was 605743 UTM Easting 5727638 UTM Northing.

**Table 1:** Effect of PDOP filtering on location accuracy for the stationary collar data.

Location type	Locations in group (%)	Location error (m)				
		Mean (sd)	50% CEP	95% CEP	99% CEP	100% CEP
All	100	4.20 (0.87)	4.1	13.9	26.0	44.4
PDOP<6	92	4.10 (0.84)	3.8	10.3	13.4	38.8
PDOP<3	46	3.96 (0.80)	3.6	8.2	9.4	26.4
3-D	45	3.66 (0.84)	3.2	8.6	10.3	21.7

Mean and circular error probable (CEP) values were calculated for log-transformations of the displacement from the measured location to the true location. The reported values have been back transformed.

**Table 2:** Effect of PDOP filtering on percentage of turning angles that can be calculated and direction of errors in measured locations.

Location type	Turning angles in group (%)	Direction of error (degrees)		
		Mean (sd)	z-value	p-value
All	100	129 (77)	0.89	>0.2
PDOP<6	94	42 (80)	0.02	>0.5
PDOP<3	25	140 (79)	0.29	>0.5
3-D	27	8 (79)	0.61	>0.5

**Figure 2:**
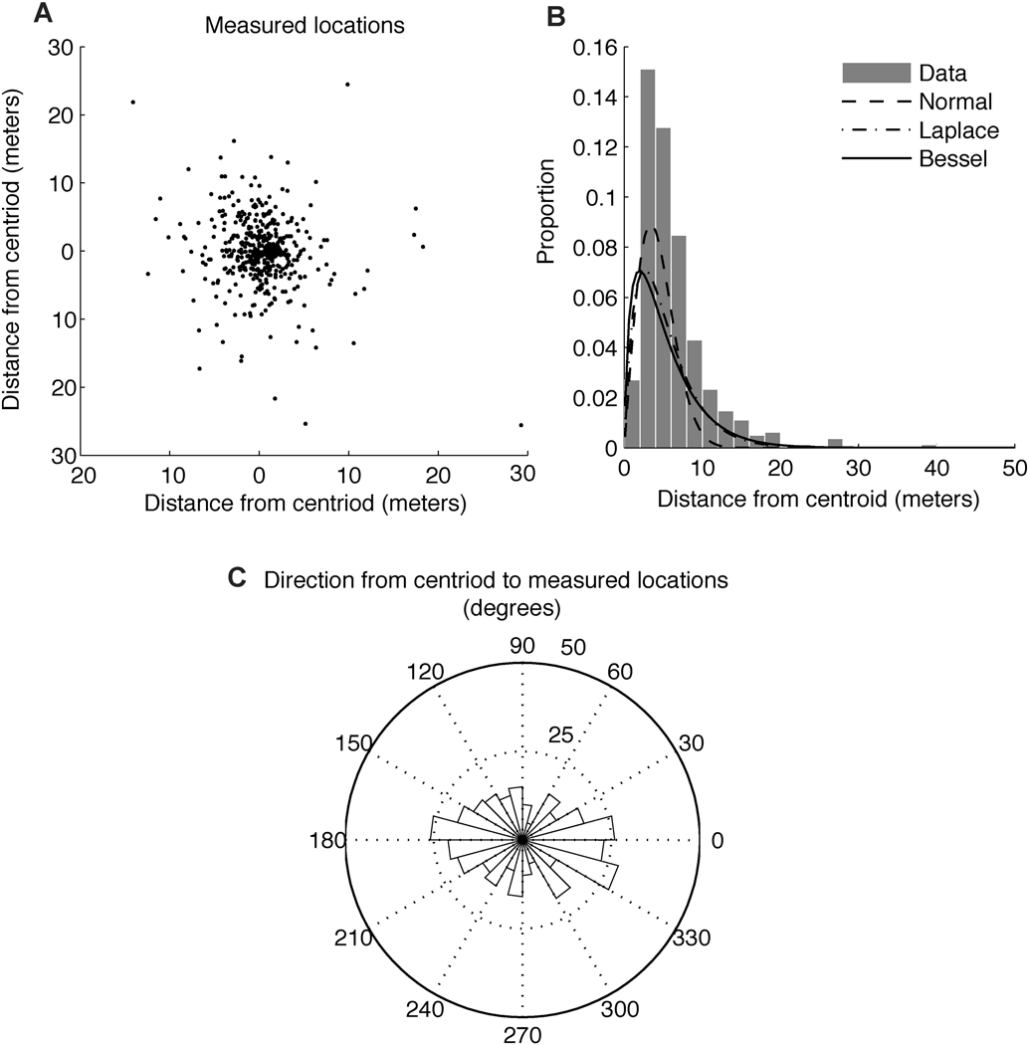
Estimating the distribution of measurement error from the stationary collar experiment. (A) GPS data collected from the stationary Lotek 2200 GPS collar. Only locations with PDOP<6 were retained (n = 406). (B) The distance from each measured location to the centroid was used to determine the best-fit distribution of GPS measurement error. Data is shown as the grey bars (in two meter intervals), the Bessel distribution (*ρ* = 0.303) is shown as the solid line, the Laplace (*β* = 2.59) is the dash-dot line, and the Normal distribution (*σ* = 4.80) is the dashed line. (C) Frequency of directions from the centroid to the measured location. There is no consistent bias in the direction of location errors (Table 2).

The best-fit parameter estimates for each candidate GPS error distribution were: Normal: *σ* = 4.80, Laplace: *β* = 2.59 and Bessel: *ρ* = 0.303 (Figure 2). All three distributions had only one parameter and the AIC values were 4867.3, 4674.6 and 4671.1 respectively. The lowest AIC value was for the Bessel distribution. The best-fit parameter estimate for the Bessel distribution implied that one error standard deviation was 4.08 meters (see Text S1).

### Case study: wolf movement data

**Figure 3:**
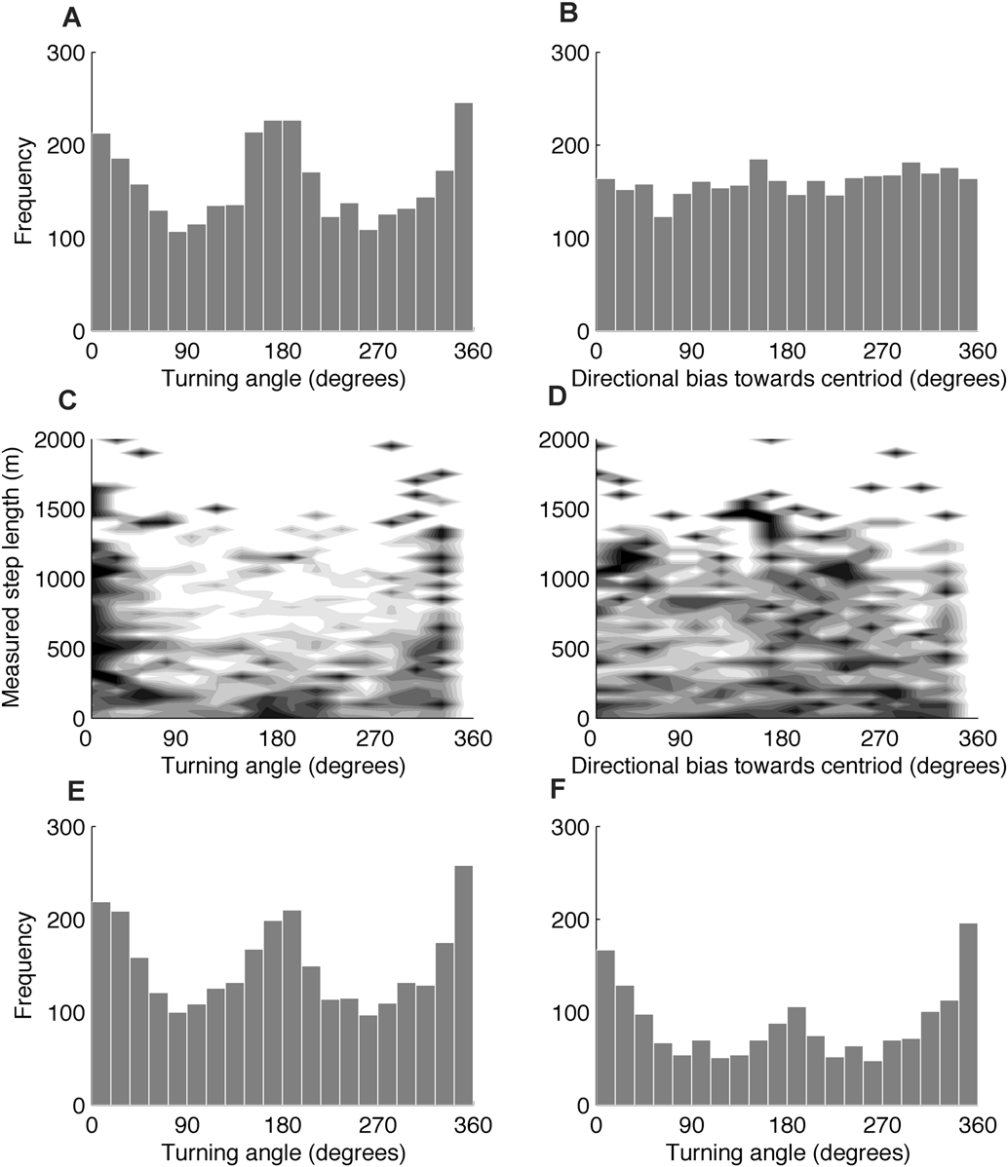
GPS data collected from a gray wolf (*Canis lupus*). GPS data were collected every 15 minutes from wolf 77 near Ya Ha Tinda Ranch, Alberta, from January 2–February 20, 2004. All locations with PDOP>5 were removed. (A) The histogram shows turning angles of 180° are most frequent (n = 3210). (B) The histogram of measured directional biases where the fixed point in the environment is the centroid of the measured locations (n = 3211). (C) The normalized relative frequency of turning angles as a function of step length is shown in grey scale with black indicating a high relative frequency. The distribution is normalized for each measured step length to correct for different number of observations for measured step lengths in the data. This panel shows the 180° turning angles occur mostly at short step lengths. These short step lengths are abundant in the data, which is why there is a high frequency of 180° turning angles shown in (A). (D) The normalized relative frequency for directional biases as a function of step length. The histogram of turning angles when only (E) 3-D fixes are retained (n = 3033) and (F) measured step lengths of >20 meters are retained (n = 1745). Increasing the accuracy of measured locations by removing all 2-D fixes has no effect. Simulation results (Table 3) suggest for measured step lengths <20 meters the pattern of 180° turning angles may be due to measurement error. The pattern of 180° turning angles is not present when only measured step lengths >20 meters are considered.

I obtained 4513 locations with a 96% fix rate from the Lotek 3300 GPS collar attached to the gray wolf in the Ya Ha Tinda area. I removed all locations with PDOP>5. This left 3786 locations corresponding to a fix rate of 80%. From these data, I calculated 3210 turning angles and 3211 directional biases with respect to the centroid of the recorded wolf locations (597372 UTM Easting, 5735289 UTM Northing). Measured locations recovered from the gray wolf showed a high frequency of 180° turning angles (Figure 3A). However, most of these 180° turning angles occurred when the wolf moved only a short distance (Figure 3C). The distribution of directional biases from the gray wolf data was approximately uniform (Figure 3B) and there was no relationship between directional bias and step length (Figure 3D). Retaining only the 3-D fixes (n = 3033) improved the accuracy of locations but did not affect the distribution of measured turning angles (Figure 3E). In contrast, retaining only measured step lengths >20 meters (n = 1745; as is suggested by Table 3) eliminated the pattern of 180° turning angles.

**Table 3:** Detection of the true turning angle.

ρ	True step length cutoff (95% CI)	Measured step length cutoff for various levels of certainty.					
		95%		75%		50%	
		Error sd	meters	Error sd	meters	Error sd	meters
0.2	1.55 (1.29, 1.94)	4.41	27.3	2.60	16.1	1.70	10.5
0.25	1.58 (1.41, 1.82)	4.38	21.7	2.58	12.8	2.02	10.0
0.3	1.62 (1.45, 1.94)	4.39	18.1	2.59	10.7	2.42	9.9
0.35	1.61 (1.41, 1.98)	4.50	15.9	2.83	10.0	2.83	10.0
0.4	1.66 (1.41, 1.98)	4.49	13.9	3.23	10.0	3.23	10.0
0.45	1.62 (1.29, 1.94)	4.47	12.3	3.63	10.0	3.63	10.0
0.5	1.64 (1.21, 2.02)	4.52	11.2	4.04	10.0	4.04	10.0

### Measured turning angles and directional biases for stationary animals

**Figure 4:**
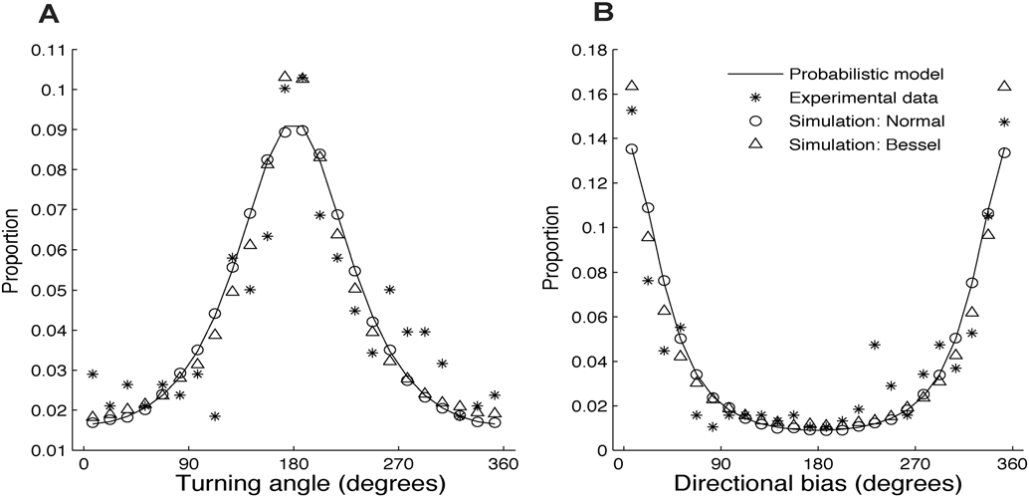
Distributions of measured (A) turning angles and (B) directional biases for stationary animals. The proportion of measured (A) turning angles and (B) directional biases for each 15° interval from 0° to 360° for the stationary collar data (*, n = 406), the probabilistic models (Equations 6 and 7, solid line), and computer simulations with Normal (*σ* = 4.80, ○) and Bessel (*ρ* = 0.303, Δ) with *M* = 0 where 100,000 measured turning angles and directional biases were generated.

From the 421 measured locations recovered from the stationary GPS collar experiment, I calculated 379 measured turning angles and 380 measured directional biases. Using the V test, I determined that the measured turning angle data were unimodal with a mean turning angle of 180° (*u* = 8.67, *p*<0.0005) and the directional biases data were unimodal with a mean equal to 0° (*u* = 13.8, *p*<0.0005). The turning angle and directional bias data from the stationary GPS collar experiment are shown as * in Figure 4. I calculated that the probability density of measured turning angles for a stationary animal and a bivariate normal distribution of measurement error was,
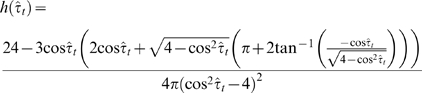
(6)(see Text S1 for full details). This probability density function has a maximum at 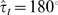 (Figure 4, solid line). For a stationary animal located at the bias point, I calculated that the probability density of measured directional biases was,
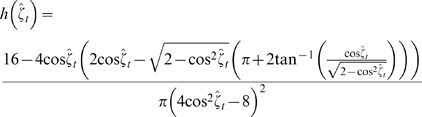
(7)(see Text S1 for full details). This probability density function has a maximum at 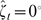 (Figure 4, solid line). I compared Equations 6 and 7 to the data from the stationary GPS collar experiment and results for the Monte Carlo simulations for stationary animals where I assumed both a Normal and Bessel distribution of measurement error. The proportion of angles in each 15° interval for the stationary GPS collar, the probabilistic model (Equations 6 and 7) and the computer simulations for stationary collars were in close agreement (Figure 4).

### Measured turning angles and directional biases for a range of step lengths

**Figure 5:**
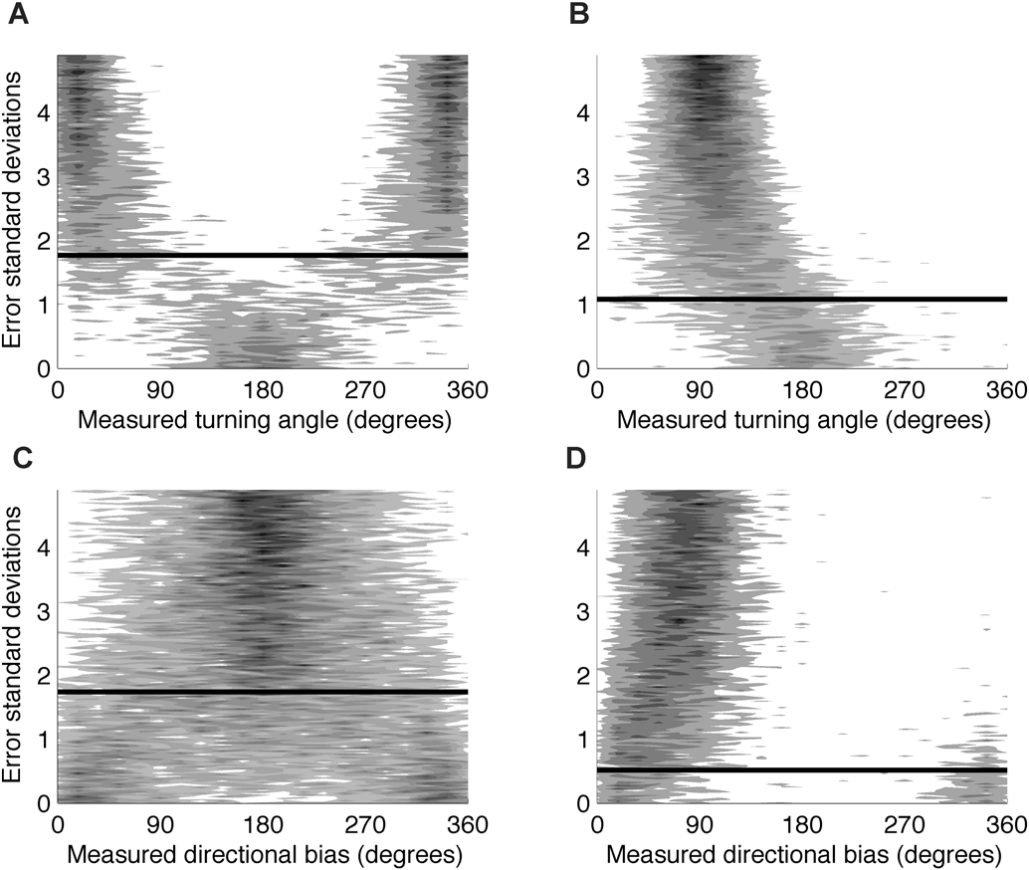
The effect of step length on turning angle and directional bias distributions. The solid line shows the minimum true step length for which the true turning angle or directional bias could be detected. (A) The distribution of measured turning angles where the true turning angle was 0°. Dark colors show more frequent observations. The solid line at 1.76 error standard derivations shows the minimum step length where the null hypothesis that the mean of the measured distribution of turning angles was not equal to the true turning angle was rejected using the V test. For the distribution of measurement error used in this simulation, this corresponds to 7.2 meters. (B) The true turning angle is 90°. The solid line at 1.08 error standard deviations is the minimum true step length where the true turning angles could be detected. This corresponds to 4.4 meters. (C) The distribution of directional biases where the true directional bias was 180°. The minimum error standard deviation where the true directional bias could be detected was 1.74. This corresponds to 7.1 meters. (D) The true directional bias was 90° and the minimum error standard deviation where the true directional bias could be detected was 0.51. This corresponds to 2.1 meters. For all simulations step lengths from 0 to 1.35 error standard deviations by 6.7×10^−4^ error standard deviations were simulated. For each step length one hundred turning angles were generated. The distance from the true location to the measured location was modeled as a Bessel distribution with ρ = 0.303.

At shorter step lengths the distribution of measured turning angles was predominated by error with a strong signal of 180° turning angles, but as step length increases the distribution becomes unimodal with mean equal to the true turning angle (Figure 5A). The minimum step length required to reject the hypothesis that the mean of the measured turning angles was not equal to 0° was 1.76 error standard deviations. This was affected by the true turning angle and for a true turning angle of 90° the minimum step length was 1.08 error standard deviations (Figure 5B). The effect of step length on measured directional bias was similar. When the true directional bias was 180°, a step length of at least 1.74 error standard deviations was needed for the true directional bias to stand apart from the error signal (Figure 5C). For a true directional bias of 90° this minimum step length was 0.51 error standard deviations (Figure 5D).

Tables 3 and 4 describe the minimum measured step length the receiver moved so that the true tuning angle or directional bias could be detected. For ρ = 0.3 there is a 95% chance that a measured step length of 18.1 meters is observed when the true step length is 1.62 error standard deviations (6.6 meters): the minimum true step length needed to detect a true turning angle of 0°. To be 50% certain the true step length threshold is exceeded, a measured step length of 9.9 meters is required. Similar results for a range of ρ values and for directional biases are provided in Tables 3 and 4.

**Table 4:** Detection of the true directional biases.

ρ	True step length cutoff (95% CI)	Measured step length cutoff for various levels of certainty.					
		95%		75%		50%	
		Error sd	meters	Error sd	meters	Error sd	meters
0.2	2.36 (1.78, 3.39)	4.36	27.0	2.58	16.0	1.62	10.0
0.25	1.88 (1.41, 2.42)	4.44	22.0	2.63	13.0	1.62	8.0
0.3	1.56 (1.21, 2.18)	4.36	18.0	2.67	11.0	1.70	7.0
0.35	1.38 (0.85, 1.98)	4.24	15.0	2.54	9.0	1.70	6.0
0.4	1.25 (0.97, 1.62)	4.52	14.0	2.58	8.0	1.62	5.0
0.45	1.14 (0.73, 1.45)	4.36	12.0	2.54	7.0	1.82	5.0
0.5	1.08 (0.81, 1.62)	4.54	11.2	2.42	6.0	1.62	4.0

## Discussion

I have shown that GPS measurement error will give rise to 180° turning angles and strong directional biases in movement data (Figure 4). This result appeared consistently in the stationary collar experiment, the probabilistic model and the stochastic model. This result alerts researchers to a false signal that could be misinterpreted as animal behavior. I parameterized the distribution of measurement error from a stationary GPS collar and ran simulations that suggested the spurious effects of measurement error are unlikely to occur for measured step lengths >20 meters. As evidence that spurious 180° turning angles may occur in animal movement data, GPS data recovered for a gray wolf showed a high frequency of 180° turning angles. This pattern may have been caused by measurement error.

**Figure 6:**
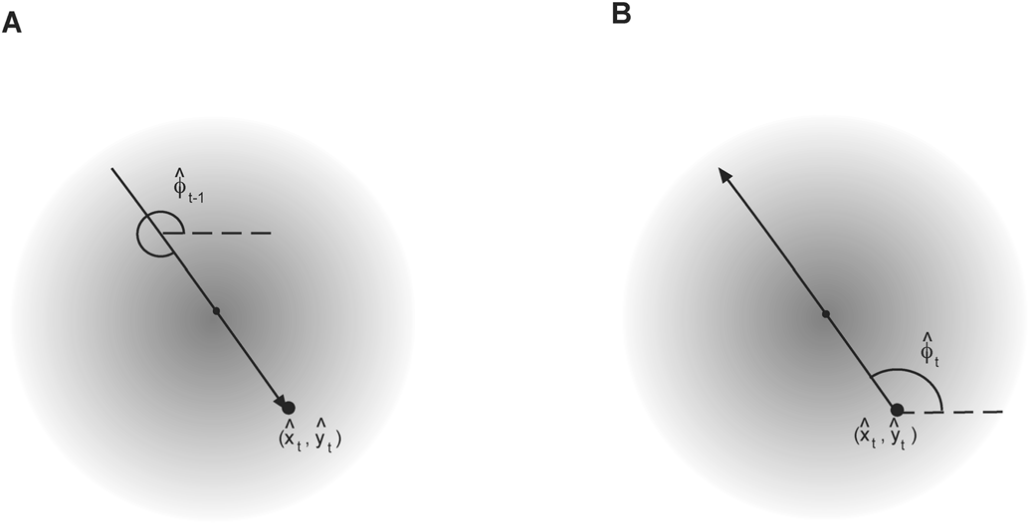
Intuitive explanation. For a stationary animal, the distribution of GPS error is centered at the same point for all time. The probability density of measured animal locations is shown in grey scale where darker shades represent higher probabilities. (A) For an animal measured at (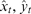) the probability the animal was measured to have come *from* the direction 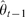 is the sum of the probabilities for all measured locations with a direction of 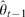. Let the most likely direction the animal is measured to come from be 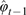. Note that 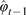 is the direction of a vector that terminates at (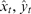) and travels through the maximum of the GPS error probability density function. (B) Using similar logic, the animal is most likely to be measured to move *away* from (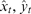) in the direction 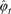. Note that 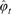 is the direction of a vector that originates at (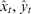) and travels through the maximum of the GPS error distribution. Turning angles are measured as 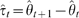. Therefore, the most likely measured turning angle is 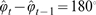 because 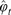 and 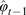 point in exactly opposite directions.

To understand intuitively why measurement error would give rise to 180° turning angles, consider a stationary animal and a symmetrical distribution of GPS measurement error with a global maximum at the animal's true location. Assume the distribution of error monotonically decreases away from the maximum. Let the animal's location be denoted as (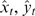). Recall that a turning angle requires three successive locations and let (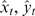) be the second of the three. The most likely direction the animal was measured to have come from must pass through the maximum of the distribution of measurement error and is denoted as 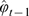 (Figure 6A). The most likely direction the animal will be measured to move to must also pass through the maximum and is denoted as 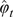 (Figure 6B). Because 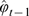 points to the location that 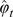 originates from, and because the distribution of GPS measurement error does not move through time, 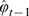 and 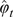 must point in exactly opposite directions. It follows that the most likely turning angle 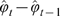 must be 180°. A similar argument can be used to explain why an animal located at the bias point that does not move is most likely to have a measured directional bias of 0°.

This research draws attention to the types of results that are signatures of measurement error. Tables 3 and 4 show that spurious 180° turning angles for measured step lengths up to 20 meters may be caused by measurement error. The results in Tables 3 and 4 are consistent with [15] who suggest that measured step lengths of >5 error standard deviations are needed for accurate estimates of measured turning angles.

The measured step length results for directional bias (Table 4) are less robust since the true step length cutoffs were generated by assuming the receiver was 0.34 error standard deviations (5 meters) from the bias point. This is quite conservative and it is likely that the extent to which spurious 0° directional biases are seen is less than the measured step lengths reported in Table 4. Another restrictive assumption is the use of the V test to detect the true turning angle and directional bias. This tests that the sample mean approximates the true mean and this does not imply the distribution of measured turning angles is a good approximation of the distribution of true turning angles. An accurate approximation of the true distribution would require a longer measured step length. The filtering method shown in Figure 3F removed all measured step lengths <20 meters. Another possible filtering method is to weight turning angles according to the measured step length. A similar approach was successfully used to resolve location accuracy by weighting locations by the known accuracy of different fix types [30]. Other possible improvements are to use activity monitors to identify and remove periods of inactivity from location data [35], [36].

If researchers wish to improve the accuracy of measured turning angle and directional bias distributions the best course of action is to increase the accuracy of measured locations. These are well documented: dense canopy cover, changes in elevation, atmospheric conditions and poor satellite geometry will all reduce location accuracy [13], [23], [27]. When the interval between GPS measurements is longer, the animal is able to move further between fixes, and more data can be retained [15], [16]. Location accuracy can be improved with differential correction [24], wide [37] and local [23] area augmentation, or real time differential GPS [23] that use carrier phase enhancement. However, following the removal of selective availability in 2000 these types of investments are less warranted [24] unless understanding fine scale movements is necessary.

For parameterization of mechanistic movement models, the objective is to accurately determine the distribution of turning angles, directional biases or step lengths and not necessarily to accurately resurrect the animal's true location, movement path or home range. This alters the performance of data filtering methods because it alters how accuracy is improved relative to the cost of data loss. Improving the accuracy of the measured turning angles and directional biases depends on the accuracy of measured locations relative to the distance that the collar moves between locations. This is complicated by the relationship between location accuracy and measured step length, since measured step lengths overestimate true step lengths and more so when location accuracy is poor [15].

The costs of removing a single location from the data are higher when turning angles are considered. Calculating a turning angle requires three successive locations and calculating a directional bias requires two successive locations. Filtering out locations can dramatically reduce the number of turning angles or directional biases that can be calculated, in much the same way as a decreased fixed rate [15]. As a further consideration, errors in measured location are uniform (but see [38], [39]) while errors in measured turning angles and directional biases are systematic.

The differences between location accuracy and turning angle accuracy are discussed to point out that studies reporting turning angles and directional biases cannot cite CEP and other measures of location accuracy to justify claims that measurement error does not affect their results. Previous studies report the effect of location error on other descriptors of animal movement: fractal dimension [14], linear feature use [32] and resource selection [40], [41]. Bootstrapping, where errors in measured locations are simulated, is a simple method to resolve the accuracy of descriptive statistics. For individual-based simulations, measurement error could be added to the recorded GPS locations, the distributions of measured turning angles and directional biases are recalculated and animal movement then simulated. Even if GPS locations are quite accurate the importance of such a procedure should not be overlooked due to the profound effect that measurement error can have on turning angle accuracy.

From the data collected from the gray wolf, I cannot conclude that the 180° turning angles (Figure 2) are behavioral because measurement error remains a likely cause. The systematic bias in measured turning angles also suggests that measurement error will produce movement with two distinct modes: 1) short step lengths with spurious 180° turning angles and 2) longer step lengths where the measured turning angles reflect the true turning angles. It is important to note that the appearance of such modes is not necessarily behavioral and could be driven by measurement error. For example, these two modes will appear if an animal moves in a straight line at a speed that is a random variable drawn form a uniform distribution. In this case the appearance to two different types of movement is spurious. To properly affirm the existence of two distinct behavioral modes the use of activity sensors [35], [36] are suggested.

GPS measurement error can have significant consequences for conservation biology because GPS data are widely used to design conservation plans. A possible consequence is that studies failing to consider measurement error may detect directed movement towards a fixed point in space when no directed movement exists. The high frequency of 180° turning angles at short step lengths is relevant to studies that identify movement states from measured turning angles and step lengths (e.g., [42], [43]). For individual-based simulation models, if the spurious effects of measurement error are not removed from turning angle distributions, movement paths will overestimate the frequency that animals return to previously visited locations and predict overly tortuous movement. Previous studies have found more tortuous movement on smaller spatial scales [10], [44] when measured step lengths are smallest relative to the variance of the distribution of GPS measurement error. A possible consequence of over predicting the tortuousity of movement is that animals are predicted to cross linear features more often than they actually do. For example, an accurate understanding of the effects of linear features on animal movement (e.g., effects of roads and seismic lines on Woodland caribou (*Rangifer tarandus*), [45], [46]) is essential for the sustainable management of wildlands. Other effects of measurement error on simulated animal movement are relative to the true animal movement. For an animal moving in a straight line, failing to remove measurement error has the effect of underestimating species range, and overestimating the use of a core range area. Core areas are often used in the delineation of protected areas [47], [48]; therefore, accurate estimation of these areas is essential for delineating appropriate ecological boundaries.

The first stage of an analysis of movement data is to perform error correction [49]. Unlike errors in measured locations, measured turning angles are affected by a systematic bias. The false biological signals observed by [14] and the dramatic drop off in the inaccuracy of measured turning angles as a function of step length [15] are probably caused by spurious 180° turning angles (Figure 5). For the most part, movement of large mammals is not biologically meaningful if the displacement between measured locations is less than 20 meters (especially because the true displacement is likely nearer to 9.9 meters; Tables 3 and 4). Therefore, for most studies improving the accuracy of measured locations is not particularly necessary, instead it is important to be mindful of interpreting results only to the accuracy possible for the equipment. One concern is that as technology advances GPS receivers will be attached to smaller animals and it will be possible to collect data with higher fix rates. Interpretation of data collected under these scenarios will need to carefully consider the effects of GPS measurement error. If there is any uncertainty as whether the receiver being used is sufficiently accurate, an important message from this work is to treat 180° turning angles with suspicion.

## Supporting Information

Text S1 Mathematical calculations to determine the: 1) error standard deviations for the Bessel distribution, 2) probability density of measured turning angles and 3) probability density of measured directional biases.
(0.10 MB PDF)
