# Supplementary material for: GPS Measurement Error Gives Rise to Spurious 180° Turning Angles and Strong Directional Biases in Animal Movement Data
Source: PLoS One. 2009 May 20;4(5):e5632. doi: 10.1371/journal.pone.0005632 (PMC2682572; doi:10.1371/journal.pone.0005632)
Supplement: Text S1 — Mathematical calculations to determine the: 1) error standard deviations for the Bessel distribution, 2) probability density of measured turning angles and 3) probability density of measured directional biases. (0.10 MB PDF) [file pone.0005632.s001.pdf]

Supplementary Material

Error standard deviations

The standard derivation of the Bessel distribution is the square root of the variance $E[r^2] - E[r]^2$ where $E[r] = \int_0^\infty rf(r)\ dr$ and $E[r^2] = \int_0^\infty r^2 f(r)\ dr$. Therefore, the standard deviation for the Bessel distribution is $\frac{\sqrt{16-\pi^2}}{2\rho}$. From the stationary GPS collar experiment the best estimate of ρ was 0.303. Therefore, from this experiment one standard error deviation is 4.08 meters. This value is close to the value of σ, the standard deviation, estimated for the normal distribution.

Deriving the probability density function of measured turning angles and directional biases

The probability density of measured turning angles and directional biases is determined through changes of variables. This approach is based on Solow (1990) who addressed a similar mathematical question under a different motivation.

Probabilistic model for turning angles

The distribution of GPS measurement error is bivariate Normal with covariance equal to zero and mean $(0, 0)$,

$$f(\hat{x}_t, \hat{y}_t) = \frac{1}{2\pi\sigma^2} e^{-\frac{\hat{x}_t^2 + \hat{y}_t^2}{2\sigma^2}}. \tag{S.1}$$

Because the animal does not move, the distribution of GPS measurement error is the same for the locations at time $t+1$ and $t+2$. The joint pdf is,

$$g_1(\hat{x}_t, \hat{y}_t, \hat{x}_{t+1}, \hat{y}_{t+1}, \hat{x}_{t+2}, \hat{y}_{t+2}) = \frac{1}{8\pi^3\sigma^6} e^{-\frac{(\hat{x}_t^2+\hat{y}_t^2)+(\hat{x}_{t+1}^2+\hat{y}_{t+1}^2)+(\hat{x}_{t+2}^2+\hat{y}_{t+2}^2)}{2\sigma^2}}. \tag{S.2}$$

The change of variables to find the measured x and y displacement is,

$$\begin{aligned} \hat{u}_t &= \hat{x}_{t+1} - \hat{x}_t, & \hat{u}_{t+1} &= \hat{x}_{t+2} - \hat{x}_{t+1}, \\ \hat{v}_t &= \hat{y}_{t+1} - \hat{y}_t, & \hat{v}_{t+1} &= \hat{y}_{t+2} - \hat{y}_{t+1}, \\ \hat{w} &= \hat{x}_t, & \hat{z} &= \hat{y}_t. \end{aligned}$$

Because the transformation of variables is one-to-one and the absolute value of the determinant of the Jacobian matrix of the change of variables, $|J|$, is non-zero, by

Theorem 8.8 in Ghahramani (2005)[1] Eq. S.2 with the change of variables is,

$$g_2(\hat{u}_t, \hat{u}_{t+1}, \hat{v}_t, \hat{v}_{t+1}, \hat{w}, \hat{z}) = \frac{1}{8\pi^3\sigma^6} e^{-\frac{\hat{w}^2 + (\hat{u}_t+\hat{w})^2 + (\hat{u}_{t+1}+\hat{u}_t+\hat{w})^2 + \hat{z}^2 + (\hat{v}_t+\hat{z})^2 + (\hat{v}_{t+1}+\hat{v}_t+\hat{z})^2}{2\sigma^2}}. \quad \text{(S.3)}$$

where the absolute value of the determinant of the Jacobian, $|J| = 1$. The six dimensional pdf (Eq. S.3) contains variables that will not be needed ($\hat{w}$ and $\hat{z}$). The marginal distribution is,

$$\begin{aligned} g_3(\hat{u}_t, \hat{u}_{t+1}, \hat{v}_t, \hat{v}_{t+1}) &= \int_{-\infty}^{\infty} \int_{-\infty}^{\infty} g_2(\hat{u}_t, \hat{u}_{t+1}, \hat{v}_t, \hat{v}_{t+1}, \hat{w}, \hat{z}) \; d\hat{w} \; d\hat{z}, \\ &= \frac{1}{12\pi^2\sigma^4} e^{-\frac{(\hat{u}_t-\hat{u}_{t+1})^2 + \hat{u}_t\hat{u}_{t+1} + (\hat{v}_t+\hat{v}_{t+1})^2 - \hat{v}_t\hat{v}_{t+1}}{3\sigma^2}}. \end{aligned} \quad \text{(S.4)}$$

I change variables so the measured direction of the move at t and $t+1$ can be determined. Using the standard trigonometric relationships,

$$\begin{aligned} \hat{u}_t &= \hat{L}_t \cos\hat{\theta}_t, & \hat{v}_t &= \hat{L}_t \sin\hat{\theta}_t, \\ \hat{u}_{t+1} &= \hat{L}_{t+1} \cos\hat{\theta}_{t+1}, & \hat{v}_{t+1} &= \hat{L}_{t+1} \sin\hat{\theta}_{t+1}, \end{aligned}$$

since the measured step length ($\hat{L}_t$) forms the hypotenuse of a right angled triangle with adjacent side of length $\hat{x}_{t+1} - \hat{x}_t$ and an opposite side of length $\hat{y}_{t+1} - \hat{y}_t$. The absolute value of the determinant of the Jacobian matrix for this change of variables is $\hat{L}_t\hat{L}_{t+1}$. With these change of variables Eq. S.4 is,

$$g_4(\hat{L}_t, \hat{L}_{t+1}, \hat{\theta}_t, \hat{\theta}_{t+1}) = \frac{\hat{L}_t\hat{L}_{t+1}}{12\pi^2\sigma^4} e^{-\frac{\hat{L}_t^2 + \hat{L}_{t+1}^2 + \hat{L}_t\hat{L}_{t+1}\cos(\hat{\theta}_t - \hat{\theta}_{t+1})}{3\sigma^2}} \quad \text{(S.5)}$$

The last change of variables is to determine the measured turning angle $\hat{\tau}_t = \hat{\theta}_{t+1} - \hat{\theta}_t$. Noting that $-\hat{\tau}_t = \hat{\theta}_t - \hat{\theta}_{t+1}$ and $\cos\hat{\tau}_t = \cos(-\hat{\tau}_t)$. Integrating out $\hat{L}_t, \hat{L}_{t+1}$ and $\hat{\theta}_t$,

$$h(\hat{\tau}_t) = \int_0^{2\pi} \int_0^{\infty} \int_0^{\infty} \frac{\hat{L}_t\hat{L}_{t+1}}{12\pi^2\sigma^4} e^{-\frac{\hat{L}_t^2 + \hat{L}_{t+1}^2 + \hat{L}_t\hat{L}_{t+1}\cos\hat{\tau}_t}{3\sigma^2}} \; d\hat{L}_t \; d\hat{L}_{t+1} \; d\hat{\theta}_t. \quad \text{(S.6)}$$

The probability densities of u_t and θ_t have previously been described in more detail in Solow (1990) where they are referred to as A_i and D_i respectively.

[1] A similar theorem can be found in most introductory probability texts under for transformations of two or more variables

Solving the turning angle integral for turning angles

In this section I solve the triple integral Eq. S.6.

$$
\begin{aligned}
h(\hat{\tau}_t) &= \int_0^{2\pi}\int_0^{\infty}\int_0^{\infty} \frac{\hat{L}_t\hat{L}_{t+1}}{12\pi^2\sigma^4} e^{-\frac{\hat{L}_t^2+\hat{L}_{t+1}^2+\hat{L}_t\hat{L}_{t+1}\cos\hat{\tau}_t}{3\sigma^2}}\ d\hat{L}_t\ d\hat{L}_{t+1}\ d\hat{\theta}_t, \\
&= \int_0^{\infty}\int_0^{\infty} \frac{\hat{L}_t\hat{L}_{t+1}}{6\pi\sigma^4} e^{-\frac{\hat{L}_t^2+\hat{L}_{t+1}^2+\hat{L}_t\hat{L}_{t+1}\cos\hat{\tau}_t}{3\sigma^2}}\ d\hat{L}_t\ d\hat{L}_{t+1}. \qquad \text{(S.7)}
\end{aligned}
$$

Integration on the plane $(\hat{L}_t, \hat{L}_{t+1})$ is performed over the first quadrant. Changing to polar coordinates, $\hat{L}_t = R\cos\beta$, $\hat{L}_{t+1} = R\sin\beta$, where $|J| = R$,

$$
\begin{aligned}
h(\hat{\tau}_t) &= \int_0^{\pi/2}\int_0^{\infty} \frac{R^3\cos\beta\sin\beta}{6\pi\sigma^4} e^{-\frac{R^2(1+\cos\beta\sin\beta\cos\hat{\tau}_t)}{3\sigma^2}} dR\ d\beta, \\
&= \frac{1}{6\pi\sigma^4}\int_0^{\pi/2}\cos\beta\sin\beta\int_0^{\infty} R^3 e^{-\frac{R^2(1+\cos\beta\sin\beta\cos\hat{\tau}_t)}{3\sigma^2}} dR\ d\beta. \qquad \text{(S.8)}
\end{aligned}
$$

Using another change of variables,

$$
\eta = \frac{R^2(1+\cos\beta\sin\beta\cos\hat{\tau}_t)}{3\sigma^2}, \qquad d\eta = \frac{2R(1+\cos\beta\sin\beta\cos\hat{\tau}_t)}{3\sigma^2} dR,
$$

$$
\begin{aligned}
h(\hat{\tau}_t) &= \frac{1}{6\pi\sigma^4}\int_0^{\pi/2}\cos\beta\sin\beta\left(\frac{3\sigma^2}{1+\cos\beta\sin\beta\cos\hat{\tau}_t}\right)^2 \frac{1}{2}\int_0^{\infty}\eta e^{-\eta} d\eta\ d\beta, \\
&= \frac{3}{4\pi}\int_0^{\pi/2}\frac{\cos\beta\sin\beta}{(1+\cos\beta\sin\beta\cos\hat{\tau}_t)^2}\ d\beta. \qquad \text{(S.9)}
\end{aligned}
$$

Let,

$$
h(\hat{\tau}_t) = \frac{3}{4\pi}\ \frac{dF}{dx}. \qquad \text{(S.10)}
$$

where $x = -\cos\hat{\tau}_t$ and,

$$
\begin{aligned}
\frac{dF}{dx} &= \int_0^{\pi/2} \frac{\cos\beta\sin\beta}{(1-x\cos\beta\sin\beta)^2}\ d\beta, \\
&= \int_0^{\pi/2} \frac{d}{dx}\frac{1}{1-x\cos\beta\sin\beta}\ d\beta, \\
&= \frac{d}{dx}\int_0^{\pi/2} \frac{1}{1-x\cos\beta\sin\beta} d\beta. \qquad \text{(S.11)}
\end{aligned}
$$

Taking the antiderivative,

$$
F(x) = \int_0^{\pi/2} \frac{1}{1-x\cos\beta\sin\beta}\ d\beta. \qquad \text{(S.12)}
$$

Using the change of variables $z = \tan\beta$, where $d\beta = dz/(1+z^2)$ and,

$$
\cos\beta\sin\beta = \frac{\cos\beta\sin\beta}{\cos^2\beta + \sin^2\beta} = \frac{\frac{\sin\beta}{\cos\beta}}{1+\left(\frac{\sin\beta}{\cos\beta}\right)^2} = \frac{z}{1+z^2}, \qquad \text{(S.13)}
$$

Substituting Eq. (S.13) and $d\beta$ into Eq. (S.12),

$$
\begin{aligned}
F(x) &= \int_0^{\infty} \frac{1}{1-\frac{xz}{1+z^2}} \frac{dz}{1+z^2}, \\
&= \int_0^{\infty} \frac{dz}{1+z^2-xz}, \\
&= \int_0^{\infty} \frac{dz}{\left(z-\frac{x}{2}\right)^2 + 1 - \frac{x^2}{4}}, \\
&= \int_0^{\infty} \frac{dz}{\left(1-\frac{x^2}{4}\right)\left[\left(\frac{z-\frac{1}{2}x}{\sqrt{1-\frac{x^2}{4}}}\right)^2 + 1\right]}. \qquad \text{(S.14)}
\end{aligned}
$$

Let,

$$
\phi = \frac{z-\frac{1}{2}x}{\sqrt{1-\frac{x^2}{4}}}, \qquad dz = \sqrt{1-\frac{x^2}{4}} d\phi,
$$

$$
\begin{aligned}
F(x) &= \frac{1}{\sqrt{1-\frac{x^2}{4}}} \int_{-\frac{x}{2\sqrt{1-\frac{x^2}{4}}}}^{\infty} \frac{d\phi}{\phi^2+1}, \\
&= \frac{1}{\sqrt{1-\frac{x^2}{4}}} \tan^{-1}\phi \Big|_{-\frac{x}{2\sqrt{1-\frac{x^2}{4}}}}^{\infty}, \\
&= \frac{\pi + 2\tan^{-1}\left(\frac{x}{\sqrt{4-x^2}}\right)}{\sqrt{4-x^2}}. \qquad \text{(S.15)}
\end{aligned}
$$

$$
\begin{aligned}
\frac{dF}{dx} &= \frac{2\left(\frac{x^2}{(4-x^2)^{3/2}} + \frac{1}{\sqrt{4-x^2}}\right)}{\sqrt{4-x^2}\left(1+\frac{x^2}{4-x^2}\right)} + \frac{x\left(\pi + 2\tan^{-1}\left(\frac{x}{\sqrt{4-x^2}}\right)\right)}{(4-x^2)^{3/2}}, \\
&= \frac{8 - x\left(2x - \sqrt{4-x^2}\left(\pi + 2\tan^{-1}\left(\frac{x}{\sqrt{4-x^2}}\right)\right)\right)}{\left(x^2-4\right)^2}. \qquad \text{(S.16)}
\end{aligned}
$$

Substituting Eq. (S.16) into Eq. (S.10) and re-substituting $x = -\cos\tau_t$,

$$
\begin{aligned}
h(\hat{\tau}_t) &= \frac{3}{4\pi}\frac{dF}{dx}, \\
&= \frac{24 - 3\cos\hat{\tau}_t\left(2\cos\hat{\tau}_t + \sqrt{4-\cos^2\hat{\tau}_t}\left(\pi + 2\tan^{-1}\left(\frac{-\cos\hat{\tau}_t}{\sqrt{4-\cos^2\hat{\tau}_t}}\right)\right)\right)}{4\pi\left(\cos^2\hat{\tau}_t - 4\right)^2}. \qquad \text{(S.17)}
\end{aligned}
$$

Probabilistic model for directional bias

The derivation and analysis of the probabilistic model for directional bias is analogous to the probabilistic turning angle model. I consider the special case where the animal is stationary and is located at the bias point where $(x_t, y_t) = (x_{t+1}, y_{t+1}) = (\Psi, \chi) = (0, 0)$. The distribution of measurement error is assumed bivariate Normal distributed (Eq. S.1). The joint probability of any pair of locations $(\hat{x}_t, \hat{y}_t)$ and $(\hat{x}_{t+1}, \hat{y}_{t+1})$ is,

$$
g_1(\hat{x}_t, \hat{x}_{t+1}, \hat{y}_{t+1}, \hat{y}_{t+1}) = \frac{1}{4\pi^2\sigma^4} e^{-\frac{\hat{x}_t + \hat{x}_{t+1} + \hat{y}_t + \hat{y}_{t+1}}{2\sigma^2}}. \qquad \text{(S.18)}
$$

I used the change of variables,

$$\begin{array}{ll}\hat{u}_t = \hat{x}_{t+1} - \hat{x}_t, & \hat{v}_t = \hat{y}_{t+1} - \hat{y}_t, \\ \hat{w}_t = \hat{x}_t, & \hat{z}_t = \hat{y}_t\end{array} \tag{S.19}$$

where $\hat{w}_t$ and $\hat{z}_t$ are the x and y distances to the bias point. With the change of variables,

$$g_2(\hat{u}_t, \hat{v}_t, \hat{w}_t, \hat{z}_t) = \frac{1}{4\pi^2\sigma^4} e^{-\frac{\hat{u}_t^2+\hat{v}_t^2+2\hat{u}_t\hat{w}_t+2\hat{v}_t\hat{y}_t+2(\hat{x}_t^2+\hat{y}_t^2)}{2\sigma^2}}. \tag{S.20}$$

I used another change of variables,

$$\begin{array}{ll}\hat{u}_t = \hat{L}_t \cos\hat{\theta}_t, & \hat{v}_t = \hat{L}_t \sin\hat{\theta}_t, \\ \hat{w}_t = \hat{M}_t \cos\hat{\Theta}_t, & \hat{z}_t = \hat{M}_t \sin\hat{\Theta}_t,\end{array}$$

where absolute value of the determinant of the Jacobian matrix is $\hat{L}_t\hat{M}_t$. The directional bias is $\hat{\zeta}_t = \hat{\Theta}_t - \hat{\theta}_t$,

$$h(\hat{\zeta}_t) = \int_0^{2\pi}\int_0^{\infty}\int_0^{\infty} \frac{\hat{L}_t\hat{M}_t}{4\pi^2\sigma^4} e^{-\frac{\hat{L}_t^2+2\hat{M}_t^2-2\hat{L}_t\hat{M}_t\cos\hat{\zeta}_t}{2\sigma^2}}\ d\hat{L}_t\ d\hat{M}_t\ d\hat{\theta}_t. \tag{S.21}$$

Solving the triple integral for directional bias

In this section I solve Eq. S.21,

$$\begin{aligned} h(\hat{\zeta}_t) &= \int_0^{2\pi}\int_0^{\infty}\int_0^{\infty} \frac{\hat{L}_t\hat{M}_t}{4\pi^2\sigma^4} e^{-\frac{\hat{L}_t^2+\hat{M}_t^2-2\hat{L}_t\hat{M}_t\cos\hat{\zeta}_t}{2\sigma^2}}\ d\hat{L}_t\ d\hat{M}_t\ d\hat{\theta}_t, \\ &= \int_0^{\infty}\int_0^{\infty} \frac{\hat{L}_t\hat{M}_t}{2\pi\sigma^4} e^{-\frac{\hat{L}_t^2+\hat{M}_t^2-2\hat{L}_t\hat{M}_t\cos\hat{\zeta}_t}{2\sigma^2}}\ d\hat{L}_t\ d\hat{M}_t, \end{aligned} \tag{S.22}$$

I consider $\hat{L}_t$ and $\hat{M}_t$ as two coordinates and integration on the plane $(\hat{L}_t, \hat{M}_t)$ is performed over the first quadrant. Changing to polar coordinates, $\hat{L}_t = R\cos\beta$ and $\hat{M}_t = R\sin\beta$, where $|J| = R$,

$$h(\hat{\zeta}_t) = \frac{1}{2\pi\sigma^4}\int_0^{\pi/2} \cos\beta\sin\beta \int_0^{\infty} R^3 e^{-\frac{R^2(3-\cos 2\beta - 2\cos\zeta_t \sin 2\beta)}{4\sigma^2}} dR\ d\beta. \tag{S.23}$$

I make another change of variables,

$$\eta = \frac{R^2(3-\cos 2\beta - 2\cos\zeta_t\sin 2\beta)}{4\sigma^2}, \quad d\eta = \frac{R(3-\cos 2\beta - 2\cos\zeta_t\sin 2\beta)}{2\sigma^2}.$$

$$\begin{aligned}
h(\hat{\zeta}_t) &= \frac{1}{\pi}\int_0^{\pi/2} \frac{4\cos\beta\sin\beta}{(3-\cos 2\beta - 2\cos\zeta_t\sin 2\beta)^2}\int_0^{\infty} \eta e^{-\eta}\; d\eta\; d\beta, \\
&= \frac{4}{\pi}\int_0^{\pi/2} \frac{\cos\beta\sin\beta}{(3-\cos 2\beta - 2\cos\zeta_t\sin 2\beta)^2}\; d\beta. \qquad \text{(S.24)}
\end{aligned}$$

Let,

$$h(\hat{\zeta}_t) = \frac{4}{\pi}\,\frac{dF}{dx}. \qquad \text{(S.25)}$$

where $x = 2\cos\zeta_t$ and,

$$\begin{aligned}
\frac{dF}{dx} &= \int_0^{\pi/2} \frac{\cos\beta\sin\beta}{(3-\cos 2\beta - x\sin 2\beta)^2}\; d\beta, \qquad \text{(S.26)}\\
&= \int_0^{\pi/2} \frac{d}{dx}\frac{1}{2(3-\cos 2\beta - x\sin 2\beta)}\; d\beta,
\end{aligned}$$

where d/dx can be taken out of the integral because the last integral is convergent for all x between -1 and 1.

$$F(x) = \int_0^{\pi/2} \frac{1}{2(3-\cos 2\beta - x\sin 2\beta)}\; d\beta. \qquad \text{(S.27)}$$

Using the double angle formula,

$$\begin{aligned}
3-\cos 2\beta - x\sin 2\beta &= 3(\cos^2\beta + \sin^2\beta) - (\cos^2\beta - \sin^2\beta) - 2x\cos\beta\sin\beta, \\
&= 2(\cos^2\beta + 2\sin^2\beta + x\cos\beta\sin\beta),
\end{aligned}$$

Furthermore,

$$2(\cos^2\beta + 2\sin^2\beta + x\cos\beta\sin\beta) = \frac{2(2z^2 - xz + 1)}{1+z^2}, \qquad \text{(S.28)}$$

where $z = \tan\beta$ and the steps used to find Eq. (S.28) are shown in Eq. (S.13). Substituting Eq. (S.28) into Eq. (S.27), completing the square and factoring the denominator,

$$\begin{aligned} F(x) &= \frac{1}{4}\int_0^\infty \frac{dz}{2z^2 - xz + 1}, \\ &= \frac{1}{8}\int_0^\infty \frac{dz}{\left(\frac{1}{2} - \frac{x^2}{16}\right)\left[\left(\frac{z-\frac{x}{4}}{\sqrt{\frac{1}{2}-\frac{x^2}{16}}}\right)^2 + 1\right]}. \end{aligned} \tag{S.29}$$

Let,

$$\phi = \frac{z - \frac{x}{4}}{\sqrt{\frac{1}{2} - \frac{x^2}{16}}}, \qquad dz = \sqrt{\frac{1}{2} - \frac{x^2}{16}}\; d\phi,$$

$$\begin{aligned} F(x) &= \frac{1}{8\sqrt{\frac{1}{2} - \frac{x^2}{16}}} \int_{-\frac{x}{4\sqrt{\frac{1}{2}-\frac{x^2}{16}}}}^{\infty} \frac{d\phi}{\phi^2 + 1}\ , \\ &= \frac{1}{8\sqrt{\frac{1}{2} - \frac{x^2}{16}}} \tan^{-1}\phi \Bigg|_{-\frac{x}{4\sqrt{\frac{1}{2}-\frac{x^2}{16}}}}^{\infty}, \\ &= \frac{\pi + 2\tan^{-1}\frac{x}{\sqrt{8-x^2}}}{4\sqrt{8-x^2}}. \end{aligned} \tag{S.30}$$

$$\begin{aligned} \frac{dF}{dx} &= \frac{16 - 2x^2 + \pi x\sqrt{8-x^2} + 2x\sqrt{8-x^2}\tan^{-1}\left(\frac{x}{8-x^2}\right)}{4\left(x^2-8\right)^2}, \\ &= \frac{16 - x\left(2x - \pi\sqrt{8-x^2} - 2\sqrt{8-x^2}\tan^{-1}\left(\frac{x}{\sqrt{8-x^2}}\right)\right)}{4(x^2-8)^2}. \end{aligned} \tag{S.31}$$

Substituting Eq. (S.31) into Eq. (S.25) and re-substituting $x = 2\cos\hat{\zeta}_t$,

$$\begin{aligned} h(\hat{\zeta}_t) &= \frac{4}{\pi}\frac{dF}{dx}, \\ &= \frac{16 - 4\cos\hat{\zeta}_t\left(2\cos\hat{\zeta}_t - \sqrt{2-\cos^2\hat{\zeta}_t}\left(\pi + 2\tan^{-1}\left(\frac{\cos\hat{\zeta}_t}{\sqrt{2-\cos^2\hat{\zeta}_t}}\right)\right)\right)}{\pi(4\cos^2\hat{\zeta}_t - 8)^2}. \end{aligned} \tag{S.32}$$

References

[1] Ghahramani S. 2005. Fundamentals of Probability with Stochastic Processes, Third Edition. Pearson Prentice Hall, New Jersey, USA.

[2] Solow A. 1990. A note on the statistical properties of animal locations. J Math Biol 29: 189-193.
